# House cricket protein hydrolysates alleviate hypertension, vascular dysfunction, and oxidative stress in nitric oxide-deficient hypertensive rats

**DOI:** 10.14202/vetworld.2024.2104-2114

**Published:** 2024-09-20

**Authors:** Weerapon Sangartit, Pisit Suwannachot, Supawan Thawornchinsombut, Gulladawan Jan-On, Orachorn Boonla, Ketmanee Senaphan

**Affiliations:** 1 Department of Physiology, Faculty of Medicine, Khon Kaen University, Khon Kaen, 40002, Thailand; 2 Division of Physiology, Faculty of Veterinary Medicine, Khon Kaen University, Khon Kaen, 40002, Thailand; 3 Department of Food Technology, Faculty of Technology, Khon Kaen University, Khon Kaen, 40002, Thailand; 4 Chulabhorn International College of Medicine, Thammasat University, Pathumthani, 12120, Thailand; 5 Thammasat University Research Unit in Physiology and Integrated Medicine, Thammasat University, Pathumthani, 12120, Thailand; 6 Department of Physical Therapy, Faculty of Allied Health Sciences, Burapha University, Chonburi, 20131, Thailand

**Keywords:** angiotensin-converting enzyme activity, house cricket protein hydrolysates, hypertension, nitric oxide deficiency, oxidative stress, vascular dysfunction

## Abstract

**Background and Aim:**

Edible insects with high protein content and bioactive peptides with health promotion against chronic disease. Deficiency of nitric oxide (NO) contributes to hypertension, a leading cause of cardiovascular diseases and death worldwide. This study assessed the antihypertensive effects of house cricket protein hydrolysates (HCPH) in NO-deficient hypertensive rats.

**Materials and Methods:**

Male Sprague-Dawley rats (n = 12/group) were hypertensive after the administration of N^ω^-nitro-L-arginine methyl ester (L-NAME) at a dose of 50 mg/kg body weight (BW)/day in drinking water for 7 weeks. The animals were then treated with HCPH (250 or 500 mg/kg BW/day) or lisinopril (Lis) (1 mg/kg BW/day) for the last 4 weeks of L-NAME administration. Blood pressure (BP), vascular function, and structural changes, endothelial NO synthase (eNOS), and p47^phox^ nicotinamide adenine dinucleotide phosphate (NADPH) oxidase protein expression in aortic tissues, plasma nitrate/nitrite, plasma angiotensin-converting enzyme (ACE) activity, and oxidative stress markers in blood and tissues were evaluated.

**Results:**

Induction of hypertension resulted in significantly elevated BP, decreased plasma nitrate/nitrite concentration, abolished vascular function, and increased vascular wall thickness. Overproduction of carotid and mesenteric superoxide, increased plasma, heart, and kidney malondialdehyde, and protein carbonyl levels, and increased plasma ACE activity were observed. Down-expression of eNOS with overexpression of p47^phox^ NADPH oxidase subunit was also found in L-NAME hypertensive rats. Oral treatment with HCPH, particularly at a dose of 500 mg/kg BW/day, significantly alleviated these alterations in a manner comparable to that of Lis.

**Conclusion:**

HCPH improved vascular function and exerted antihypertensive effects, mainly due to the improvement of NO bioavailability, reduction of oxidative stress, and inhibition of ACE.

## Introduction

Nitric oxide (NO) plays an important role in vascular homeostasis. It acts as a potent vasodilator along with other vasoactive substances, such as prostacyclin and endothelin-1, to control vascular tone [1]. NO has also been established as a major mediator controlling vascular remodeling [2, 3]. Under physiological conditions, NO is produced by endothelial NO synthase (eNOS) in the vascular endothelium [1]. Reduction in NO bioavailability refers to reduce NO production or increased NO metabolism, which results in impaired vasodilation, vascular inflammation, platelet aggregation, vascular remodeling [4, 5], and hypertension [6]. Uncoupling of eNOS in endothelial cells is the main cause of reduced NO availability, suggesting endothelial dysfunction. Excessive reactive oxygen species (ROS) play an important role in hypertensive development through increased uncoupled eNOS [7]. Nicotinamide adenine dinucleotide phosphate (NADPH) oxidase is a major source of ROS and is upregulated in the presence of hypertension [8, 9]. NADPH-oxidase-derived superoxide (O_2_^•-^) rapidly reacts with NO to form peroxynitrite (ONOO-). Both O_2_^•-^ and especially ONOO- directly oxidize 5,6,7,8-tetrahydrobiopterin, an eNOS cofactor, to form uncoupled eNOS, thereby converting eNOS to a superoxide-generating enzyme. The relationship between excessive ROS and increased endothelial dysfunction has been demonstrated in several experimental hypertension models, including renovascular hypertension, NO-deficient rats, and spontaneous hypertensive rats [9–11].

Among antihypertensive drugs, angiotensin II (Ang II) receptor blockers and angiotensin-converting enzyme inhibitors (ACEi) can attenuate vascular oxidative stress and increase NO bioavailability [12]. Recently, biomedical research has shown that some plant and animal proteins and their hydrolytic products can be useful in the protective and/or therapeutic treatment of hypertension [13–16]. Documents showing different inhibitory properties of ACE activity (ACEi) in hydrolysate proteins were reported by Vercruysse *et al*. [17]. Moreover, previous findings by Boonla *et al*. [8], Jan-On *et al*. [18], and Jan-On *et al*. [19] reported that rice bran protein hydrolysates have antihypertensive and antioxidant effects in hypertensive rat models. In addition, insect protein hydrolysates exhibited ACE-inhibitory activity after digestion with enzymes [17, 20].

Edible insects containing an emerging protein, which is an alternative source of protein, are of growing interest due to their high protein content and bioactive peptides, which promote the prevention of chronic disease [13, 21]. Several studies have suggested that cricket peptides and protein hydrolysates possess antioxidant and anti-inflammatory activities and ACE-inhibiting activities that might be useful as nutraceuticals, antihypertensive agents, or synthetic antioxidants [13, 22–24]. Most of these studies on the effects of peptides/hydrolysates from crickets were conducted in an *in vitro* model, and *in vivo* and clinical trials are needed to verify these effects.

Therefore, this study examined the beneficial effects of house cricket protein hydrolysates (HCPH) on antihypertension, endothelial dysfunction, vascular structural changes, and oxidative stress in NO-deficient hypertensive rats. The hypertensive rats were developed by administration of N^ω^-nitro-L-arginine methyl ester (L-NAME), a non-selecting NOS inhibitor, resulting in depleted NO production. This model develops arterial hypertension and is associated with vascular oxidative stress, endothelial dysfunction, and vascular hypertrophy [9, 25].

## Materials and Methods

### Ethical approval

All experimental protocols and procedures performed on animals were reviewed and approved by the Institutional Animal Care and Use Committee of Khon Kaen University (IACUC-KKU-91/63) based on the Animal Experiment Ethics of the National Research Council of Thailand.

### Study period and location

This study was conducted from May 2021 to May 2022 at the Northeast Laboratory Animal Center, Khon Kaen University, the Faculty of Medicine and the Faculty of Veterinary Medicine, Khon Kaen University, Thailand.

### Cricket protein hydrolysates (HCPH) preparation

House crickets (*Acheta domesticus*) were obtained from a cricket farm in Khon Kaen, Thailand, which has been certified for Good Agricultural Practices. Whole crickets were washed and boiled for 15 min and then soaked in 0.02% butylated hydroxyl anisole and butylated hydroxyl toluene for 30 min (4°C–5°C). The soaked crickets were vacuum packed in aluminum foil bags and then frozen in an air blast freezer (−35°C; Air Blast freezer, Rivacold Ice 13–10, Tewkesbury, Gloucestershire, UK) for 2–3 h. The frozen crickets were kept at −20°C until further use. The HCPH were prepared following the protein hydrolysis procedure [26] with slight modification by adjusting the water mixing ratio, protease concentration, and hydrolysis time at the Department of Food Technology, Khon Kaen University, and followed Good Hygienic Practice while producing HCPH. The soaked crickets were thawed and rinsed once with distilled water at a 1:2 (w/v) ratio for 1 min. The crickets were minced (5 mm; BIRO 8–22 E97, The Biro Mfg. Co., USA) and mixed with distilled water at a ratio of 1:3 (w/v) using an Ace homogenizer at 10,000 rpm for 1 min. The suspension was pre-incubated and adjusted to pH 8.0 at 55°C to optimize enzyme activity. Protease P6 (a protease derived from *Bacillus licheniformis*; E.C. 3.4.21.62) was added to start a hydrolysis reaction at a concentration of 1.5 (% v/w) ml of 100 g of minced crickets (dried basis) for 3 h. Before enzyme inactivation, the suspension was adjusted to pH 7.0 and heated (95°C–98°C) in a microwave oven for 5 min. HCPH was obtained by centrifugation (10,000× *g*, 15 min, 4°C) and lyophilization of the supernatant collected from the cooled suspension.

### Bioactive activities of HCPH

Lyophilized HCPH was dissolved in distilled water, and the supernatant was collected. The radical scavenging activity of HCPH was determined using the 2,2′-azino-bis-3-ethylbenzthiazoline-6-sulfonic acid (ABTS) assay, and the metal chelating ability of HCPH was assessed as described by Wachirattanapongmetee *et al*. [27]; the reducing ability of HCPH was assessed using the ferric reducing antioxidant power (FRAP) assay [28]. The ACE inhibitory activity of HCPH was also determined by reversed-phase high-performance liquid chromatography [29], and the amino acid compositions were analyzed as described by Association of Official Analytical Chemistry [30]. Essential amino acids in HCPH are presented in Table-1.

**Table-1:** Essential amino acid content in HCPH.

Essential amino acids	g/100 g protein
Histidine	1.85
Isoleucine	2.87
Leucine	5.7
Lysine	4.59
Methionine	1.45
Phenylalanine	2.51
Threonine	3.03
Valine	4.06

HCPH=House cricket protein hydrolysates

### Animals and experimental designs

Seventy-two adult male Sprague-Dawley rats weighing 200–220 g were obtained from Nomura Siam International Co., Ltd, Bangkok, Thailand. According to reports that estrogens are antioxidants themselves [31, 32], to avoid the influence of sex hormones in females, which might affect the results of this study, only male rats have been chosen for this study. The animals were housed in a humidity- and temperature-controlled room with a 12-h/12-h light/dark cycle in the Northeast Laboratory Animal Center, Khon Kaen University, Khon Kaen, Thailand. They were fed a standard chow diet and tap water *ad libitum* during the 1 week of acclimatization period. Then, hypertension was induced in rats by administering L-NAME (50 mg/kg body weight [BW]/day) in their drinking water for 7 weeks when age- and BW-matched rats received tap water to serve as normotensive control rats. During the last 4 weeks, rats were randomly assigned into six subgroups (12 rats per group) and administered deionized water as the HCPH vehicle daily; then, HCPH at doses of 250 and 500 mg/kg BW/day or lisinopril (Lis) at 1 mg/kg BW/day was administered by gavage, starting from week 4 to week 7 of the experiment. Accordingly, the experimental groups were composed of control+vehicle, control+HCPH500, L-NAME+vehicle, L-NAME+HCPH250, L-NAME+HCPH500, and L-NAME+Lis. The doses of HCPH were obtained from our preliminary study, which found that these doses were non-toxic and had a blood pressure (BP)-lowering effect on L-NAME hypertensive rats, and the dose of Lis was the lowest dose that reduced BP in previous studies by Zhou *et al*. [33]. BW and systolic BP (SBP) were measured weekly. A non-invasive method of tail-cuff plethysmography (IITC/Life Science Instrument model 299 and model 179 amplifier; Woodland Hills, CA, USA) was used to determine SBP in conscious rats as described by Jan-On *et al*. [18] and Senaphan *et al*. [34].

### Measurement of hemodynamic status and vascular responsiveness

At the end of the experiment, to determine baseline BP, heart rate (HR), and vascular reactivity, all rats were anesthetized through intraperitoneal injection of thiopental sodium (60 mg/kg BW), and body temperature was maintained with a heating pad throughout the measurement period. A tracheotomy was performed for spontaneous breathing. The femoral artery was cannulated with a polyethylene catheter connected to a pressure transducer for continuous monitoring of BP and HR using data acquisition software (Biopac System, CA, USA). A second polyethylene catheter was inserted into the femoral vein for the infusion of vasoactive agents. Baseline values of BP and HR were monitored for 10 min and after obtaining stable baseline data, vascular reactivity was assessed by testing the vascular responses to vasoactive agents, including the vasoconstrictor (Ang II; 0.1, 0.3, 1.0 nmol/kg BW), the endothelium-dependent vasodilator acetylcholine (ACh; 3, 10, 30 nmol/kg BW) and the endothelium-independent vasodilator sodium nitroprusside (SNP; 1, 3, 10 nmol/kg BW). Each vasoactive agent was infused stepwise at 5-min intervals. Finally, rats were sacrificed by overdose of the anesthetic drug. Blood samples and organs (heart, liver, and kidney) were collected for the assay of oxidative stress biomarkers and plasma ACE activity. The carotid and mesenteric arteries were rapidly excised from the rats to assess the rate of superoxide (O_2_^•-^) production. The thoracic aorta was rapidly collected to detect the protein expression of the p47^phox^ NADPH oxidase subunit and eNOS. Morphometric analysis of the vascular wall was also performed.

### Western blot analysis

Aortic tissue homogenates were prepared to determine the protein expression of p47^phox^ NADPH oxidase subunit and eNOS according to method described by Boonla *et al*. [8]. The densities of the interested proteins were visualized and captured using an ImageQuant™ 400 imager (GE Healthcare Life Science, Piscataway, NJ, USA), with ß-actin used as the housekeeping protein.

### Histology of the thoracic vessels

The thoracic aortas of eight rats per group were used for morphometric analysis. In brief, the thoracic aorta was excised, cleaned of connective tissue, and fixed with 4% phosphate-buffered paraformaldehyde; the vessels were then processed following standard histological methods. Serial sections of 5-μM thickness were stained with hematoxylin and eosin (Bio-Optica Milano SpA., Milano, Italy). Images of the stained sections were captured using a digital microscope camera (Nikon DS-Ri1 Camera, Nikon Instruments Inc., Melville, NY, USA). The medial cross-sectional area (CSA), aortic wall thickness, media-to-lumen ratio (M/L), and lumen area were analyzed as described by Boonla *et al*. [10].

### Oxidative stress biomarkers and plasma ACE activity

Vascular superoxide (O_2_^•-^) production was assessed in isolated carotid and mesenteric arteries using a lucigenin-enhanced chemiluminescence method as described by Nakmareong *et al*. [35]. Malondialdehyde (MDA), a marker of lipid peroxidation, and a protein carbonyl marker, a marker of oxidizing protein damage, were measured in plasma and organs (heart, liver, and kidney) as described by Senaphan *et al*. [34]. The concentration of nitrate and nitrite in the plasma is recognized as the oxidative products of NO and is used as an index of NOS activity was then determined as described by Nakmareong *et al*. [35]. Plasma ACE activity was evaluated by using the O-phthalaldehyde-chromogenic reaction as described by Boonla *et al*. [10].

### Statistical analysis

Data are presented as mean ± standard error of the mean, and n refers to the number of rats. The statistical differences between groups were assessed using one-way analysis of variance and the Student-Newman-Keuls *post hoc* tests. p < 0.05 was considered statistically significant.

## Results

### Amino acid composition, ACE inhibition, and antioxidant activity of HCPH

The major essential amino acids in HCPH are valine, leucine, lysine, threonine, and isoleucine (Table-1). HCPH exhibits free radical-scavenging, antioxidant, and ACE inhibitory activities. The ABTS value of HCPH was 33.60 μg Trolox/100 μg bovine serum albumin (BSA). The FRAP value of HCPH was equivalent to 3.39 μg Trolox/1000 μg BSA, and the metal-chelating ability of HCPH was 63.19 μg ethylenediaminetetraacetic acid/1000 μg BSA. In ACE inhibition, HCPH inhibited ACE activity by 44%, and the half-maximal inhibitory concentration value of HCPH was 3.45 mg/mL.

### Therapeutic effect of HCPH on SBP in hypertensive rats

**Figure-1:**
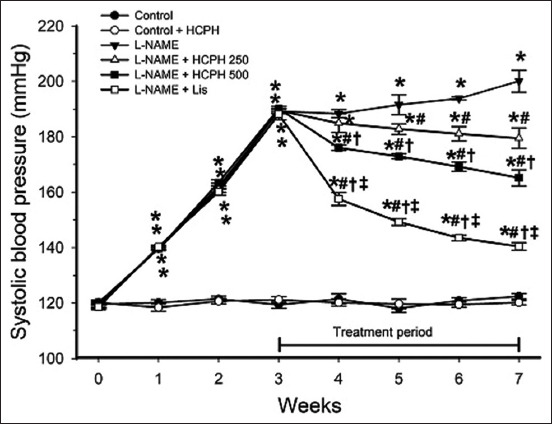
Effects of HCPH on systolic blood pressure measured by tail-cuff plethysmography once weekly throughout the experiment. L-NAME=*N*^ω^-nitro-L-arginine methyl ester, HCPH=House cricket protein hydrolysates, Lis=Lisinopril. Data are expressed as mean ± SEM, (n = 10/group), *p < 0.05 versus control, ^#^p < 0.05 versus L-NAME group, ^†^p < 0.05 versus L-NAME + HCPH250 group, and ^‡^p < 0.05 versus L-NAME + HCPH500 group.

A significant increase in SBP in rats treated with L-NAME was observed starting from 1 week after L-NAME administration until the end of the experiment (p < 0.05 compared with normal controls, Figure-1). Treatment of rats with HCPH 250 or 500 mg/kg BW/day for 4 weeks significantly reduced SBP in a dose-dependent manner. The reduction in SBP in L-NAME rats treated with Lis, which was a positive control, was higher than that in L-NAME rats administered with HCPH 250 or 500 mg/kg (p < 0.05, Figure-1).

### Therapeutic effects of HCPH on direct BP and HR in hypertensive rats

**Figure-2:**
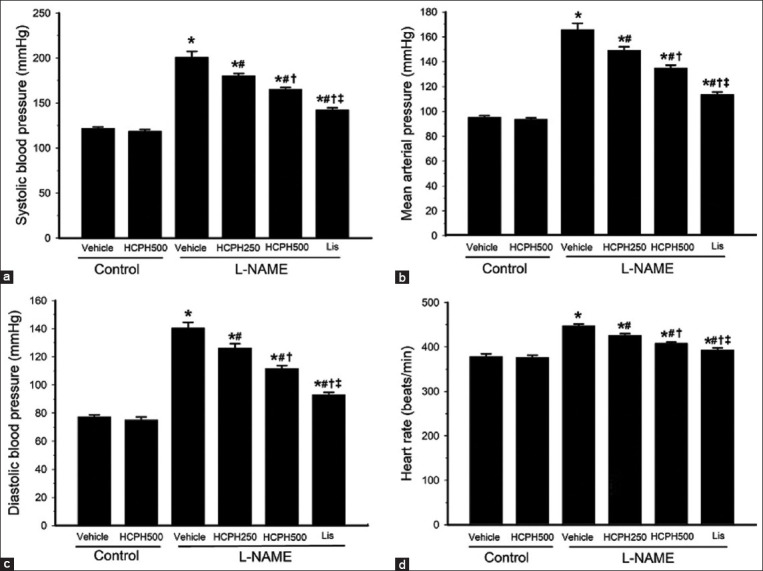
Effects of HCPH on (a) systolic blood pressure, (b) mean arterial pressure, (c) diastolic blood pressure, and (d) heart rate at the end of the experiment. L-NAME=N^ω^-nitro-L-arginine methyl ester, HCPH=House cricket protein hydrolysates, Lis=Lisinopril. Data are expressed as mean ± SEM, (n = 10/group), *p < 0.05 versus control, ^#^p < 0.05 versus L-NAME group, ^†^p < 0.05 versus L-NAME + HCPH250 group, and ^‡^p < 0.05 versus L-NAME + HCPH500 group.

The induction of hypertension in L-NAME-treated rats resulted in significantly increased SBP, mean arterial pressure (MAP), diastolic BP, and HR compared with the normal control rats (p < 0.05; Figures-2a–d). Treatment with HCPH250 and HCPH500 significantly decreased these alterations in a dose-dependent manner (p < 0.05). Lis exerted a greater effect than HCPH250 and HCPH500 on the reduction of these parameters (p < 0.05, Figures-2a–d).

### Therapeutic effects of HCPH on vascular responsiveness in hypertensive rats

**Figure-3:**
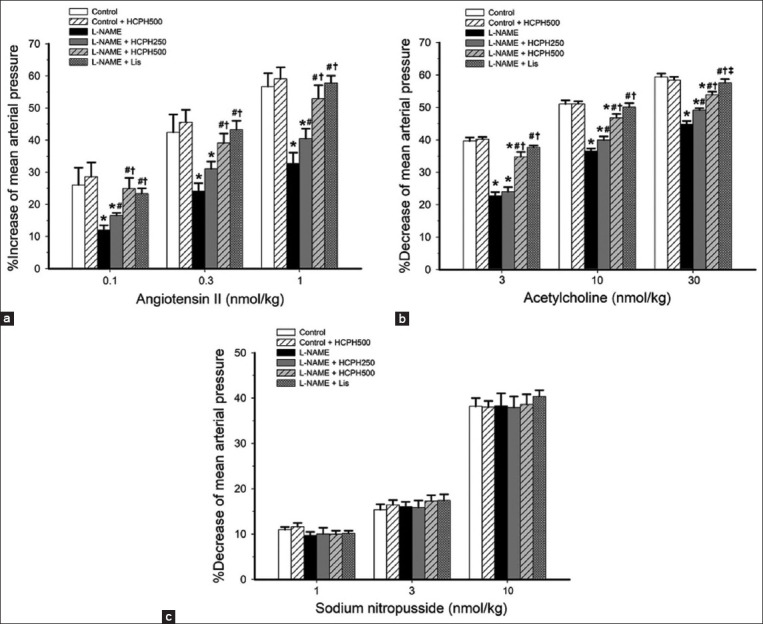
Effects of HCPH on mean arterial pressure changes induced by (a) angiotensin II, (b) acetylcholine, and (c) sodium nitroprusside in all experimental groups. L-NAME=N^ω^-nitro-L-arginine methyl ester, HCPH=House cricket protein hydrolysates, Lis=Lisinopril. Data are expressed as mean ± SEM, (n = 10/group), *p < 0.05 versus control, ^#^p < 0.05 versus L-NAME group, ^†^p < 0.05 versus L-NAME + HCPH250 group, and ^‡^p < 0.05 versus L-NAME + HCPH500 group.

Vascular reactivity was determined by observing the difference in the MAP changes after the infusion of Ang II, ACh, and SNP with baseline values. The L*-*NAME-treated rats showed a significant decrease in Ang II-induced vasoconstriction compared with the control rats (p < 0.05). Treatment with HCPH250, HCPH500, and Lis significantly restored vasoconstriction in hypertensive rats (p < 0.05, Figure-3a), suggesting that HCPH administration improves vascular smooth muscle contraction in NO-deficient rats.

The administration of L*-*NAME in rats significantly reduced ACh-induced vasodilation compared with the findings in normal control rats (Figure-3b). The percentage decreases in MAP were significantly increased in L*-*NAME-treated rats after treatment with HCPH250 or HCPH500 in a dose-dependent manner (p < 0.05, Figure-3b). Moreover, the vascular responses to ACh in the L*-*NAME+Lis group were higher than those in the L*-*NAME+HCPH250 and L*-*NAME+HCPH500 groups (p < 0.05, Figure-3b). These results imply that HCPH significantly reverses the impairment of endothelial-dependent vasodilation of ACh in NO-deficient rats. There were no significant differences in endothelium-independent vasodilators induced by SNP between the experimental groups (Figure-3c). These results suggest that L*-*NAME induces endothelial dysfunction by altering the ability of blood vessels to generate NO but not smooth muscle relaxation. Therefore, the optimal NO concentration was determined by the induced vasodilation in rats treated with L-NAME.

### Therapeutic effects of HCPH on p47^phox^ and eNOS protein expression in hypertensive rats

**Figure-4:**
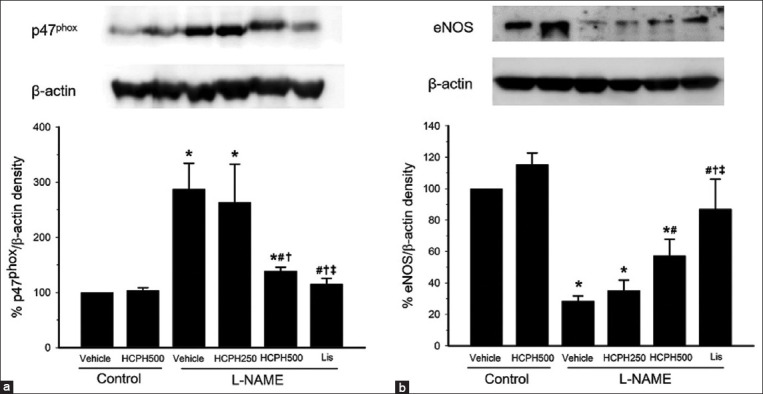
Densitometric analysis of (a) p47^phox^ and (b) eNOS protein expression in the thoracic aorta in all experimental groups. L-NAME=N^ω^-nitro-L-arginine methyl ester, HCPH=House cricket protein hydrolysates, Lis=Lisinopril, eNOS=Endothelial nitric oxide synthase. Data are expressed as mean ± SEM; (n = 4/group), *p < 0.05 versus control, ^#^p < 0.05 versus L-NAME group, ^†^p < 0.05 versus L-NAME + HCPH250 group, and ^‡^p < 0.05 versus L-NAME + HCPH500 group.

Oxidative stress is induced by increased O_2_^•-^ production and/or decreased NO production. Figure-4a presents p47^phox^ protein expression in the thoracic aorta, which is a source of O_2_^•-^ production. p47^phox^ was significantly up-regulated in rats treated with L-NAME compared with normal control rats. p47^phox^ was significantly down-regulated in L*-*NAME+HCPH500 compared with the L-NAME group (p < 0.05, Figure-4a). Figure-4b shows eNOS protein expression in the thoracic aorta, which is the source of NO synthesis. eNOS was significantly downregulated in rats treated with L-NAME compared with normal control rats. Interestingly, eNOS was significantly upregulated in L*-*NAME+HCPH500 rats compared with the L-NAME group. Lis better improved oxidative stress than HCPH, as indicated by decreased p47^phox^ expression and increased eNOS expression in L*-*NAME-treated rats (p < 0.05, Figure-4b).

### Therapeutic effects of HCPH on the arterial structures of hypertensive rats

**Figure-5:**
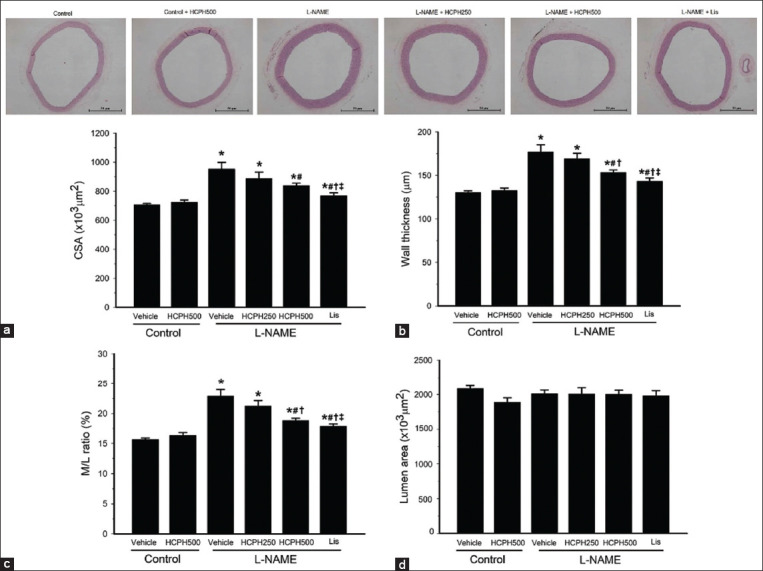
The top panel shows representative photographs of the aortic sections (40×) stained with hematoxylin and eosin. Scale bar = 50 μm. Effects of HCPH on the vascular (a) cross-sectional area, (b) wall thickness, (c) media/lumen ratio, and (d) lumen area of thoracic aorta in all experimental groups. L-NAME=N^ω^-nitro-L-arginine methyl ester, HCPH=House cricket protein hydrolysates, Lis=Lisinopril, CSA=Cross-sectional area, M/L ratio=Media-to-lumen ratio. Data are expressed as mean ± SEM; (n = 6-8/group), *p < 0.05 versus control, ^#^p < 0.05 versus L-NAME group, ^†^p < 0.05 versus L-NAME + HCPH250 group, and ^‡^p < 0.05 L-NAME + HCPH500 group.

The vascular structures of the thoracic aortas in all rats are presented as CSA, wall thickness, M/L ratio, and lumen area. Figure-5 shows the significant increase in the CSA, wall thickness, and M/L ratio in L-NAME-treated rats compared with control rats (p < 0.05, Figures-5a–c). There were no significant changes in the lumen area in the experimental groups (Figure-5d). This suggests that the vascular wall is a eutrophic remodeling type. The L-NAME rats treated with HCPH500 and Lis showed significantly improved vascular remodeling, as indicated by decreased CSA, wall thickness, and M/L ratio (p < 0.05, Figures-5a–c).

### Therapeutic effects of HCPH on oxidative stress markers and plasma ACE activity in hypertensive rats

Oxidative stress markers in this study were vascular O_2_^•-^ production, MDA, and protein carbonyl in plasma and tissue. Table-2 presents the significant increase in vascular O_2_^•-^ production, MDA content, and carbonyl content in L-NAME compared with the normal control group (p < 0.05, Table-2). The L-NAME rats treated with HCPH250, HCPH500, and Lis exhibited reduced oxidative stress, as shown by a decrease in vascular O_2_^•-^ production, MDA, and protein carbonyl and an increase in plasma nitrate/nitrite (p < 0.05, Table-2). Moreover, plasma ACE activity was significantly higher in the L-NAME group than in the normal control group. The L-NAME+HCPH250 and L-NAME+HCPH500 groups had lower plasma ACE levels than the L-NAME controls (p < 0.05, Table-2). In addition, L-NAME animals treated with Lis had significantly reduced plasma ACE activity to normal levels (Table-2).

**Table-2:** Effects of HCPH on oxidative stress markers, antioxidants, and plasma angiotensin-converting enzyme activity in all experimental groups.

Parameters	Control	Control + HCPH 500	L-NAME	L-NANE + HCPH 250	L-NAME + HCPH 500	L-NAME + Lis
Carotid superoxide production (count/mg dry weight/min)	56.8 ± 6.4	58.6 ± 6.1	155.9 ± 14.4*	124.7 ± 9.3*	89.1 ± 11.9^*#†^	79.6 ± 6.6^*#†^
Mesenteric superoxide production (count/mg dry weight/min)	73.8 ± 13.3	81.7 ± 7.8	160.7 ± 26.6*	138.02 ± 10.7*	102.4 ± 12.1^#†^	93.7 ± 10.1^#†^
Malondialdehyde						
Plasma (mM)	6.46 ± 0.79	6.55 ± 0.99	14.68 ± 1.72*	13.24 ± 1.93*	9.98 ± 0.64^*#†^	7.71 ± 0.58^#†‡^
Heart (mmol/mg protein)	4.76 ± 0.18	4.75 ± 0.19	6.52 ± 0.13*	6.06 ± 0.14^*#^	5.42 ± 0.16^*#†^	5.04 ± 0.10^#†‡^
Liver (mmol/mg protein)	4.82 ± 0.27	4.78 ± 0.20	5.23 ± 0.13	5.12 ± 0.31	4.75 ± 0.09	5.07 ± 0.16
Kidney (mmol/mg protein)	4.28 ± 0.13	4.36 ± 0.08	5.61 ± 0.21*	4.88 ± 0.13^*#^	4.53 ± 0.21^#^	4.55 ± 0.10^#^
Protein carbonyl (nmol/mg protein)						
Plasma	1.71 ± 0.06	1.64 ± 0.06	2.17 ± 0.08*	1.93 ± 0.08^#^	1.76 ± 0.05^#^	1.85 ± 0.08^#^
Heart	7.32 ± 0.60	6.82 ± 0.35	13.58 ± 1.29*	10.90 ± 1.12^*#^	7.95 ± 0.61^#†^	7.87 ± 0.83^#†^
Liver	7.30 ± 0.17	7.19 ± 0.08	7.25 ± 0.18	7.57 ± 0.26	7.49 ± 0.09	7.37 ± 0.20
Kidney	6.20 ± 0.42	5.55 ± 0.19	11.61 ± 0.86*	10.51 ± 0.27*	9.79 ± 0.62*	10.13 ± 0.64*
Plasma nitrate/nitrite (mM)	13.10 ± 0.63	12.82 ± 0.27	6.95 ± 0.69*	8.68 ± 0.15^*#^	9.49 ± 0.20^*#†^	9.69 ± 0.29^*#†^
Plasma ACE activity (mU/mL)	58.5 ± 3.09	54.8 ± 3.69	130.7 ± 9.60*	103.8 ± 3.94^*#^	92.9 ± 5.94^*#†^	65 ± 6.44^#†‡^

Data are mean ± standard error of mean; (n = 10/group),

* p < 0.05 versus control,

# p < 0.05 versus L-NAME group,

† p < 0.05 versus L-NAME+HCPH250 group, and

‡ p < 0.05 versus L-NAME+HCPH500 group. L-NAME=N^ω^-nitro-L-arginine methyl ester, HCPH=House cricket protein hydrolysates, Lis=Lisinopril, ACE-activity=Angiotensin converting enzyme-activity

## Discussion

The finding of this study revealed that HCPH exerted a dose-dependent antihypertensive effect in NO-deficient hypertensive rats. The antihypertensive effect of HCPH is related to improvements in hemodynamic status, restoration of vascular responsiveness, alleviation of arterial structural changes, and oxidative stress in hypertensive rats. The plausible mechanisms of these effects could be mediated through the downregulation of p47^phox^ NADPH oxidase, upregulation of eNOS protein expression, increased plasma nitrate/nitrite levels, inhibition of ACE activity, and the antioxidant effects of HCPH.

Consistent with Jan-On *et al*. [19], this experiment found that chronic treatment with L-NAME (50 mg/kg BW/day) for 7 weeks exhibited a blockade of NO production, induced hypertension, and endothelial dysfunction, as shown by an attenuated vascular response to the endothelium-dependent vasodilator ACh and reduced plasma nitrate/nitrite levels and decreased aortic eNOS protein expression. However, in this study, L-NAME treatment attenuated the vascular response to the vasoconstrictor Ang II, which is incompatible with the results of rats treated with L-NAME for 3 weeks, which showed an increased vascular response to vasoconstrictive agents [35]. On the other hand, our results are consistent with a previous study on rats by Nakmareong [36] that received L-NAME for 5 weeks and reported reduced response to Ang II. In addition, hyporeactivity to the vasoconstrictor Ang II was reported in the isolated aortic rings of L-NAME hypertensive rats [37]. The difference in vascular responses to vasoconstrictor agents might be explained by the chronic inhibition of NO synthesis, leading to downregulation of the contractile signaling pathway and was associated with a decrease in the extracellular Ca^2+^ content of vascular smooth muscle cells [37]. Furthermore, long-term inhibition of NO synthesis might trigger counter-regulatory mechanisms to compensate for the continued increase in vascular tone, thereby causing vascular fatigue and consequently decreasing the contractile response to vasoconstrictor agents [38, 39].

Moreover, in this study, an increased O_2_^•-^ production both in large and small arteries was also found in L-NAME hypertensive rats. This was associated with the upregulation of aortic p47^phox^ NADPH oxidase expression, an important source of enzymatic systems capable of producing ROS in endothelial cells in L-NAME hypertensive rats which is consistent with previous studies by Boonprom *et al*. [9] and Jan-On *et al*. [18]. The enhancement of O_2_^•-^ production in this study is attributed to a reduction in NO bioavailability, causing endothelial dysfunction and oxidative stress in L-NAME hypertensive rats, as indicated by elevated MDA and protein carbonyl concentrations in plasma, kidney, and heart tissues.

After treatment with HCPH or Lis for 4 weeks, current experiments found HCPH in a dose-dependent manner or Lis, causing improved hemodynamic status by lowering BP and HR, and restored endothelial function by an increased vascular response to ACh and Ang II, especially in high-dose HCPH (500 mg/kg). The plausible mechanism underlying these effects is related to enhanced NO synthesis and reduced vascular O_2_^•-^ production through the upregulation of eNOS protein and downregulation of p47^phox^ NADPH oxidase. Furthermore, the BP-lowering effect of HCPH was associated with a reduction in plasma ACE activity. Previous studies by Jan-On *et al*. [18] and Jan-On *et al*. [40] reported that Lis, an ACEi or protein hydrolysate that possesses an ACE inhibitory effect, reduced BP in L-NAME hypertensive rats, consistent with the current results, which showed that plasma ACE activity in L-NAME hypertensive rats decreased after treatment with HCPH or Lis for 4 weeks. In addition, chronic inhibition of NO synthesis through L-NAME has been reported to activate ACE activity and Ang II, which is a potent activator of NADPH oxidase, contributes to ROS production and oxidative stress [19, 41]. These findings are consistent with the current findings. Although the Ang II levels in L-NAME hypertensive rats were not measured, we found that plasma ACE activity and oxidative stress were increased. HCPH, especially at high doses, or Lis treatment reduced plasma ACE activity, alleviating oxidative stress by decreasing O_2_^•-^ production and suppressing lipid and protein oxidation by reducing MDA and protein carbonyl concentrations. These findings agree with previous studies by Hall and Liceaga [22], Hall *et al*. [23], and De Matos *et al*. [42], which reported the anti-hypertensive effects of cricket peptides and protein hydrolysates through ACE inhibition and antioxidant activity.

It has been established that a common consequence of hypertension in NO-deficient hypertensive rats is vascular remodeling [9, 19]. The blockade of NO production and oxidative stress induces vascular remodeling through NADPH oxidase activation, leading to excessive production of O_2_^•-^ promoting vascular cell growth and proliferation, and increased collagen deposition and vascular inflammation [9, 19, 43]. In accordance with previous studies [9, 19], we found that the thoracic wall of NO-deficient hypertensive rats induced by L-NAME for 7 weeks showed vascular remodeling as indicated by increased vascular wall thickness, M/L ratio, and media CSA of the thoracic wall without a significant change in lumen area. These changes were restored after treatment with high-dose HCPH or Lis for 4 weeks. The plausible mechanisms of these effects of HCPH and Lis may be associated with increased NO synthesis through the upregulation of eNOS protein expression and alleviation of oxidative stress through downregulation of p47^phox^ NADPH oxidase, which leads to a decrease in vascular O_2_^•-^ production and increased NO bioavailability. The results of this study are in line with a previous study by Ahn *et al*. [44], which found that cricket extract and cricket peptides have anti-oxidative effects in diabetic mice, in a *Caenorhabditis elegans* model [24], and in a demonstrated anti-atherosclerosis or anti-inflammation effect in high-fat diet fed rats [45]. Furthermore, the ACE-inhibitory effect of HCPH might be one of the mechanisms that alleviated vascular structural changes in hypertensive rats because there is evidence that Ang II plays a crucial role in the initiation of vascular inflammation and remodeling [46]. Nevertheless, further studies are required to elucidate the mechanism by which HCPH mitigates vascular remodeling in NO-deficient hypertensive rats.

Many studies [13, 17, 20, 22, 23, 47] have reported that insect-derived peptides exhibit ACE inhibitory activity. A previous study by Vercruysse *et al*. [47] demonstrated the ACE inhibitory peptides (Ala-Val-Phe) that were purified from insect protein hydrolysates and confirmed to have antihypertensive activity in spontaneously hypertensive rats. Recently, an *in vitro* study suggested that cricket protein hydrolysates contain potent peptides with potential ACE-inhibiting capacity, and the sequences of ACE-inhibitory peptides such as YKPRP, PHGAP, and VGPPQ were identified [23]. Enzyme selection during enzymatic protein hydrolysis is a key factor in the generation of ACE inhibitory peptides [48]. Different enzymes, such as alcalase, thermolysin, flavourzyme, neutrase, protamex, acid protease, and alkaline protease, have been applied to generate ACE-inhibiting hydrolysates [48]. In addition, combining different enzymes or conventional hydrolysis techniques, such as microwave and ultrasound treatment, has been used to achieve increased bioactivity of ACE inhibition [48]. In this study, we applied alkaline protease to hydrolyze cricket protein and generated HCPH, which could decrease ACE activity and BP in L-NAME hypertensive rats. However, the bioactive peptides in HCPH have not been isolated and identified. Therefore, further investigation is needed to identify potent peptides that inhibit ACE activity in HCPH.

Although the cricket is normally eaten in several countries for a long time and is also commercially bred for use as a food source for animals such as amphibians, birds, and reptiles [49], their safety as a food source is still a concern and requires attention. In this study, we used HCPH extracts from *A. domesticus*, which are extensively reared for human consumption within the most commonly consumed and studied species [50, 51]. A previous study evaluated the toxicity of cricket powder from *A. domesticus* in cells and mice using a genotoxicity test (5000 μg/mL for cells and up to 2000 mg/kg for mice) and oral toxicity study for 14 or 90 consecutive days (up to 3000 mg/kg). Their results suggested that in both tests, cricket powder from *A. domesticus* did not have any toxic effect [49]. In addition, *A. domesticus* was recently approved by the Commission Implementing Regulation (EU) 2022/188 as a novel food ingredient [50]. Together with the results of this study, cricket powder or HCPH from *A. domesticus* can be used as a new food, nutrient, or nutraceutical.

Some limitations of this study should be considered because a possible allergenic response is a major concern with regard to insect consumption. We did not perform an allergenicity test for HCPH before use, and even this study did not detect any allergenic responses in rats. However, allergies associated with HCPH consumption must be carefully considered and addressed before use in other animal species such as companion animals or humans. In addition, Lis was used as a positive control. These results showed that HCPH (500 mg/kg) decreased BP and all harmful effects caused by L-NAME less than Lis. Lis (1 mg/kg) did not restore all changes in L-NAME hypertensive rats to normal levels. Therefore, these results could be the basis of further studies, and it would be interesting to examine combined therapy with HCPH and Lis, which might be more effective than either HCPH or Lis alone. Furthermore, this study reported the efficacy of HCPH in an *in vivo* model of hypertension. However, the biologically active peptides derived from ingested foods in rodents, may be different from humans. Therefore, the antihypertensive effect observed in rats must be determined in humans.

## Conclusion

The findings of this study suggest that oral treatment with HCPH exerts effects against hypertension, endothelial dysfunction, oxidative stress, and vascular structural changes in NO-deficient hypertensive rats. The anti-hypertensive effect of HCPH might be mediated by an increase in NO bioavailability and the alleviation of oxidative stress through the activation of eNOS protein expression, downregulation of p47^phox^ NADPH oxidase, the reduction of O_2_^•-^ production, and the inhibition of ACE activity. These findings support the notion that protein hydrolysates from cricket has antioxidant and antihypertensive properties. This agent might be used as a nutraceutical and natural alternative to treat oxidative stress-related hypertension. Further research is essential to isolate potent peptides with ACE inhibitory properties in HCPH, and clinical trials are needed to validate the use of HCPH as a dietary supplement for reducing BP in humans.

## Authors’ Contributions

KS: Conceived and designed the study. KS, WS, and GJ: Conducted the experiment, data and sample collection, and laboratory works. WS, KS, OB, and PS: Performed data analysis. ST: Prepared the HCPH. KS, WS, and OB: Wrote the original draft of the manuscript. KS, ST, and PS: Reviewed and revised the manuscript. All authors have read, reviewed, and approved the final manuscript.
